# PANoptosis-based molecular subtyping and HPAN-index predicts therapeutic response and survival in hepatocellular carcinoma

**DOI:** 10.3389/fimmu.2023.1197152

**Published:** 2023-06-15

**Authors:** Fei Song, Cheng-Gui Wang, Jia-Zhen Mao, Tian-Lun Wang, Xiao-Liang Liang, Chen-Wei Hu, Yu Zhang, Lu Han, Zhong Chen

**Affiliations:** ^1^ Department of Hepatobiliary Surgery, Affiliated Hospital of Nantong University, Medical School of Nantong University, Nantong, China; ^2^ Jiangsu Vocational College of Medicine, Yancheng, China

**Keywords:** hepatocellular carcinoma (HCC), PANoptosis, immune characteristics, drug sensitivity, YWHAB, prognostic index

## Abstract

**Background:**

Hepatocellular carcinoma (HCC) is a highly prevalent and fatal cancer. The role of PANoptosis, a novel form of programmed cell death, in HCC is yet to be fully understood. This study focuses on identifying and analyzing PANoptosis-associated differentially expressed genes in HCC (HPAN_DEGs), aiming to enhance our understanding of HCC pathogenesis and potential treatment strategies.

**Methods:**

We analyzed HCC differentially expressed genes from TCGA and IGCG databases and mapped them to the PANoptosis gene set, identifying 69 HPAN_DEGs. These genes underwent enrichment analyses, and consensus clustering analysis was used to determine three distinct HCC subgroups based on their expression profiles. The immune characteristics and mutation landscape of these subgroups were evaluated, and drug sensitivity was predicted using the HPAN-index and relevant databases.

**Results:**

The HPAN_DEGs were mainly enriched in pathways associated with the cell cycle, DNA damage, Drug metabolism, Cytokines, and Immune receptors. We identified three HCC subtypes (Cluster_1, SFN+PDK4-; Cluster_2, SFN-PDK4+; Cluster_3, SFN/PDK4 intermediate expression) based on the expression profiles of the 69 HPAN_DEGs. These subtypes exhibited distinct clinical outcomes, immune characteristics, and mutation landscapes. The HPAN-index, generated by machine learning using the expression levels of 69 HPAN_DEGs, was identified as an independent prognostic factor for HCC. Moreover, the high HPAN-index group exhibited a high response to immunotherapy, while the low HPAN-index group showed sensitivity to small molecule targeted drugs. Notably, we observed that the YWHAB gene plays a significant role in Sorafenib resistance.

**Conclusion:**

This study identified 69 HPAN_DEGs crucial to tumor growth, immune infiltration, and drug resistance in HCC. Additionally, we discovered three distinct HCC subtypes and constructed an HPAN-index to predict immunotherapeutic response and drug sensitivity. Our findings underscore the role of YWHAB in Sorafenib resistance, presenting valuable insights for personalized therapeutic strategy development in HCC.

## Graphical Abstract

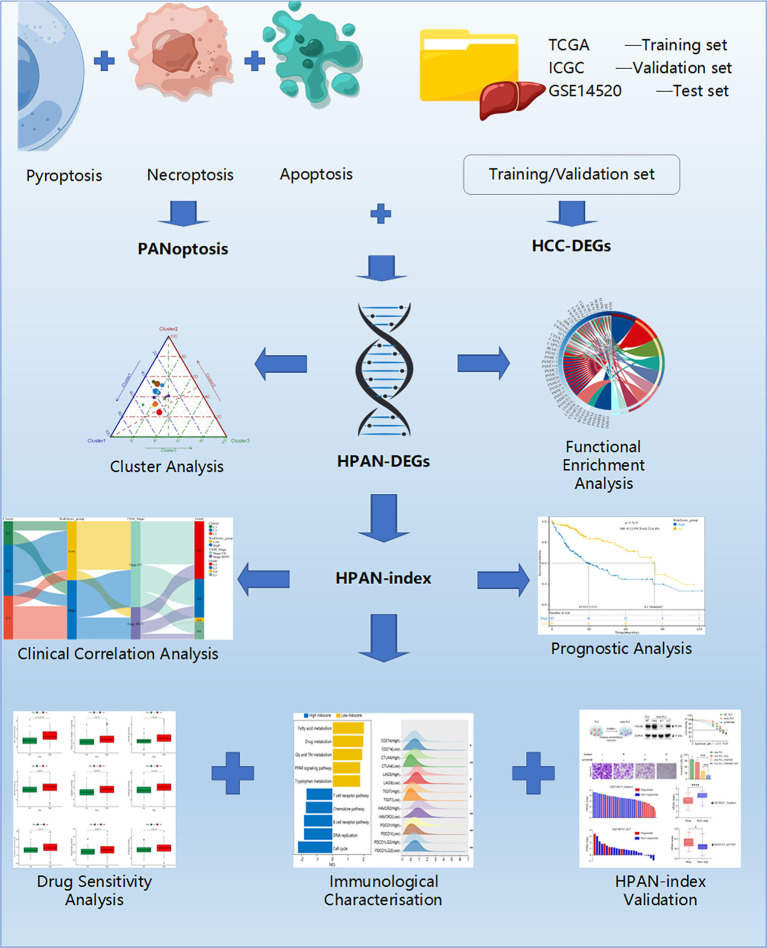

## Introduction

Hepatocellular carcinoma (HCC), the world’s third most common solid malignant tumor, is currently facing a critical situation (1, 2). In 2020, HCC was responsible for the second-highest cancer-related deaths worldwide, following only lung cancer (3, 4). Unfortunately, this proportion has steadily increased yearly, ranked third in 2017 and fourth in 2015 (5). The specific heterogeneity of liver cancer patients, coupled with the limited number of tests and treatments available, is a significant factor contributing to this situation (6, 7). Although surgery remains the primary treatment for primary liver cancer, it is only accessible to a small percentage of patients.

Moreover, even after successful surgical treatment, some patients remain at risk of recurrence and metastasis for several years thereafter (8). Despite remarkable advancements in the treatment of various cancers, such as lung cancer and melanoma, the effectiveness of immunotherapy in treating hepatocellular carcinoma (HCC) has been unsatisfactory. Specifically, the objective response rate (ORR) of PD-1 immune checkpoint blockers (ICBs) for advanced HCC hovers around 15%, which is not sufficient (9, 10). Similarly, sorafenib, a small representative molecule targeted drug, has limited survival benefits for patients with advanced liver cancer due to tumor resistance (11, 12). As such, there is an urgent need to accurately and effectively screen patients suitable for ICBs or targeted drug sensitivity, enabling them to receive the most suitable treatment.

In recent years, scholars have defined a novel cell death pathway, PANoptosis (‘P’ for Pyroptosis; ‘A’ for Apoptosis; ‘N’ for Necroptosis), which has been defined by scholars (13–15). This pathway involves the activation of a cytoplasmic multiprotein complex called PANoptosome, which can trigger multiple forms of programmed cell death, including pyroptosis, apoptosis, and necroptosis (16–18). The dysregulation of PANoptosis has been associated with various human diseases, including autoinflammatory diseases, cancer, and infectious and metabolic disorders. Some biomarkers associated with PANoptosis, including NLRP3, caspase-1 for pyroptosis, ZBP1, IRF1, caspase-8 for apoptosis, and RIPK3/RIPK1 for necroptosis, have shown considerable benefits in suppressing cancer (19–21). For instance, IRF1 functions in both myeloid and epithelial cells to counteract AOM/DSS-induced colorectal tumorigenesis, while RIPK3 activation in colon cancer cells leads to increased cytokine expression in the tumor microenvironment, contributing to robust cytotoxic anti-tumor immunity (19, 22). It is widely recognized that cell death resistance is a hallmark feature of hepatocellular carcinoma, and tumor cells have developed various strategies, such as the loss of TP53 tumor suppressor function, to limit apoptosis, which also plays a pivotal role in the failure of traditional cancer treatment (23, 24).

Cancer immunotherapy is a promising modality that stimulates the immune system to eliminate cancer cells with minimal side effects by modulating inherent immunosurveillance (25). Although some immune checkpoint blockade (ICB) therapies, particularly anti-PD-L1/PD-1, have shown clinical efficacy for patients with advanced stages of cancer, the objective response rate and survival benefits remain limitation (26, 27). One important reason for this is the inability of ICBs to induce programmed cell death (PCD) is essential for organismal development, host defense against pathogens, and maintaining homeostasis (28). However, resistance to PCD has been shown to promote tumor development, highlighting the need for novel PCD-based cancer therapies (29, 30). As a pivotal inflammatory PCD pathway, PANoptosis possesses critical features of pyroptosis, apoptosis, and necroptosis, which cannot be accounted for by any of these three PCD pathways alone (31). PANoptosis triggers systematic inflammation by releasing pro-inflammatory intracellular contents, making it a promising avenue for solid tumor immunotherapy (32). Thus, a deeper understanding of the mechanisms underlying PANoptosis can offer new opportunities to develop effective strategies for hepatocellular carcinoma immunotherapy.

There is a gap in current research on the role of PANoptosis in HCC. In the present study, we conducted a Consensus-Cluster-Plus analysis to identify three subgroups based on differentially expressed genes associated with PANoptosis and HCC (HPAN_DEGs). We then investigated these subgroups’ immune profiles and mutational landscape and constructed a PANoptosis risk score model (HPAN-index) for HCC. The HPAN-index can be used to grade the prognostic risk of HCC and to predict response to immunotherapy and chemotherapy drugs. Furthermore, we developed an integrated scoring nomogram to improve prognostic stratification and predictive accuracy for individual patients. Finally, we validated the drug response in different HPAN-index groups using public databases and *in vitro* trials, highlighting the enormous clinical potential of our findings in improving personalized decision-making for immunotherapy in HCC (Graphical abstract of the study).

## Methods

### Data acquisition and preprocessing

Data were obtained from the Cancer Genome Atlas (TCGA) in the training set, whereas the validation set sample data was sourced from the International Cancer Genome Consortium (ICGC) database. The test set sample data (GSE14520) was acquired from the Gene Expression Omnibus (GEO) database (33–36). Additionally, the GSE109211 cohort was used as a dataset for Sorafenib resistance validation, and the GSE100797 and GSE93157 cohorts were used as datasets for immunotherapy sensitivity evaluation (37–39). Please refer to **Supplementary Table 1** for detailed information on the data.

To generate the PANoptosis gene list, we merged the gene lists of pyroptosis, apoptosis, and necroptosis while eliminating any redundant genes. Specifically, the pyroptosis gene list was retrieved from the Reactome pathway database, while the apoptosis gene list was integrated from three separate gene lists obtained from the AmiGO2, Reactome, and KEGG pathway databases, respectively (40, 41). Furthermore, the necroptosis gene list was sourced from the AmiGO2 database. After compiling the individual gene lists, a total of 277 non-redundant genes were identified and included in subsequent analyses (**Supplementary Figure 1**).

### Identification of differentially expressed genes associated with HCC and PANoptosis

In our study, differential analysis was performed using the “limma” package (version 3.40.6) to identify genes differentially expressed between normal and cancer groups based on the data obtained from the TCGA and ICGC databases. To this end, we obtained the expression spectrum dataset and utilized the “lmFit” function to perform multiple linear regression. Next, the “eBays” function was utilized to calculate moderated t-statistics, F-statistics, and log-odds of differential expression via empirical Bayes moderation of standard errors directed toward an anticipated value. Subsequently, we identified the significance of variations for each gene (42). The selection criteria for identifying differentially expressed genes (DEGs) were P<0.05 and |log2FC|>1.5.

### Unsupervised clustering of HCC-PANoptosis-related model genes

We performed consensus clustering analysis to identify unknown hepatocellular carcinoma (HCC) subtypes using the “Consensus-Cluster-Plus” package and model genes (43). The clustering was executed with a 1-Pearson correlation distance, and 80% resampling of the sample, and the process was repeated ten times. Empirical cumulative distribution function plots were utilized to determine the optimal number of clusters.

### Functional enrichment analysis

To carry out GO and KEGG functional enrichment analyses, we employed the R packages “org.Hs.eg.db” and “clusterProfiler” (version 3.14.3). Initially, genes were annotated with GO terms using “org.Hs.eg.db” and mapped to a background set. Subsequently, the “clusterProfiler” package was utilized for GO and KEGG enrichment analyses, obtaining gene set enrichment results. In both cases, the minimum and maximum gene set sizes were set at 5 and 5000, respectively. We acquired the latest KEGG pathway gene annotations through the KEGG REST API and mapped them to a background set. Statistical significance was determined by a P value of < 0.05 and a false discovery rate (FDR) of < 0.25 for both analyses (44).

For Gene Set Enrichment Analysis (GSEA), we obtained subset collections from the Molecular Signatures Database to evaluate the relevant pathways and molecular mechanisms based on gene expression profiles and phenotype grouping (41). We performed 1000 permutations to obtain statistically significant results by P value of < 0.05 and FDR of < 0.25.

### Somatic mutation analysis

To evaluate somatic mutations and assess tumor mutation burden (TMB), we utilized the “maftools” R package (45, 46). Somatic mutation data was obtained from the TCGA database and analyzed to identify non-synonymous somatic mutations. We then calculated TMB scores by dividing the number of non-synonymous somatic mutations by the total size of the genome in megabases.

### Immune landscape analysis

The Tumor Immune Dysfunction and Exclusion (TIDE) framework is a computational tool that evaluates the potential for tumor immune evasion using gene expression profiles of cancer samples (47, 48). TIDE scores computed for each tumor sample serve as biomarkers to predict the response to immune checkpoint blockade, including anti-PD1 and anti-CTLA4, across different cancer types. We employed five algorithms to evaluate immune cell infiltration in the tumor microenvironment: TIMER, EPIC, xCELL, CIBERSORT, and MCPcount (49, 50). These algorithms enable a comprehensive evaluation of the immune cell landscape in the tumor microenvironment.

### Chemotherapy response and small-molecule drugs

Data from the Genomics of Drug Sensitivity in Cancer (GDSC) database were analyzed to predict chemotherapy response in HCC patients (51). The half-maximal inhibitory concentration (IC_50_) calculated using the “pRRophetic” R package was used to indicate response to chemotherapy drugs (52). To identify potential new targets for HCC treatment, the gene expression profiles of high-risk and low-risk patient groups were compared using the Connectivity Map (CMap) reference dataset (53). Specifically, differentially expressed genes were identified and ranked based on their enrichment in the CMap dataset. A drug was considered a potential target if the enrichment score was between -1 and 0 and the adjusted p-value was less than 0.05.

### Survival analysis and machine learning

We established a Lasso regression model using the “glmnet” package and utilized 10-fold cross-validation to select the optimal Lambda value, enhancing the interpretability and predictive accuracy of the model. The Lambda value of 0.0024 was optimal for minimizing the cross-validation error. We determined the coefficients of each gene using multivariate Cox analysis and generated the final regression model with the selected Lambda value. At the Lambda value of 0.0024, IRAK1, PSMD11, CHMP2A, PTRH2, SFN, YWHAB, PSMD3, TP53BP2, and PSMA4 were identified as the most important genes for predicting STATUS, with a calculated scoring formula of:.


$$HPANi=\sum_{i=1}^{9}\beta i*Ei$$

We initially divided patients into two groups based on the risk coefficient value using the percentile (50%) and classified them as either the high HPAN-index or low HPAN-index groups. Subsequently, we used the “survfit” function in the R software package “survival” to analyze the prognostic differences between the two groups. The log-rank test method was employed to evaluate the significance of the prognostic differences between the samples in different groups.

### Cell proliferation, western blot, and invasion assays

The inhibitory effect of sorafenib on cell growth was assessed using the Cell Counting Kit-8 (CCK-8, Dojindo Kumamoto, Japan). Cells were plated at a density of 5,000 cells per well in 96-well plates. Following an initial 8-hour incubation period, the cells were treated with sorafenib at the prescribed doses or left untreated for 48 hours. Specific monitoring steps can be referred to in the instructions provided by the CCK-8 kit. Western blotting was carried out in the manner previously mentioned (54).

For the Matrigel invasion experiment, 1:8 diluted Matrigel matrix gel coating from Corning (ME) was applied to the chamber. DMEM plates without FBS were utilized to inject 2 × 10^6^ cells per group. DMEM supplemented with 20% fetal bovine serum was added to the lower chamber, while mitomycin C was administered to the upper chamber to prevent cell proliferation. After a 48-hour incubation, the submembrane surface-invading tumor cells were fixed with 4% methanol and stained with 0.1% crystal violet. Each sample was counted across × 100 microscopic fields. All assays were performed in triplicate to ensure reliability.

### Construction of sorafenib-resistant cell lines and RNA interference

The resis-PLC cells were generated from PLC cells using a protocol involving continuous exposure to increasing concentrations of sorafenib, followed by stepwise selection (55). The cells were collected every 3-4 days, passaged, and cultured in DMEM media containing progressively higher concentrations of sorafenib until they could grow steadily in its presence.

Small interfering RNA (siRNA) oligonucleotides specific to the target gene were used to knockdown expression. Cells were transfected with siRNA oligonucleotides using Lipofectamine 3000 (Invitrogen) according to the manufacturer’s protocol. The siRNA sequences for the YWHAB gene were as follows: si-1 5’-GCTGAATTGGATACGCTGAAT-3’, si-2 5’-CCAATGCTACACAACCAGAAA-3’, and si-NC 5’-UUCUCCGAACGUGUCACGUdTdT-3’. The RNA duplexes were synthesized by Genomeditech (Shanghai, China). Knockdown efficiency was assessed by western blotting.

### Statistical analysis

Statistical evaluations were conducted utilizing R software (v.4.1.0). Continuous variables were displayed as mean ± standard deviation (SD), while categorical variables were shown as frequency (percentage). The Student’s t-test or Wilcoxon test examined differences between two groups concerning continuous variables contingent upon data normality assumptions. The chi-square or Fisher’s exact test was applied to categorical variables based on anticipated frequency counts. A two-sided P-value below 0.05 was deemed statistically significant across all tests. The Kaplan-Meier technique was implemented for survival assessment, and the log-rank test was adopted to compare group variations. Multivariate survival analysis utilized Cox proportional hazards regression. All analyses were conducted by a professional statistician with over 5 years of experience.

## Results

### Identification and functional analysis of PANoptosis-associated differentially expressed genes for HCC

**Figure 1:**
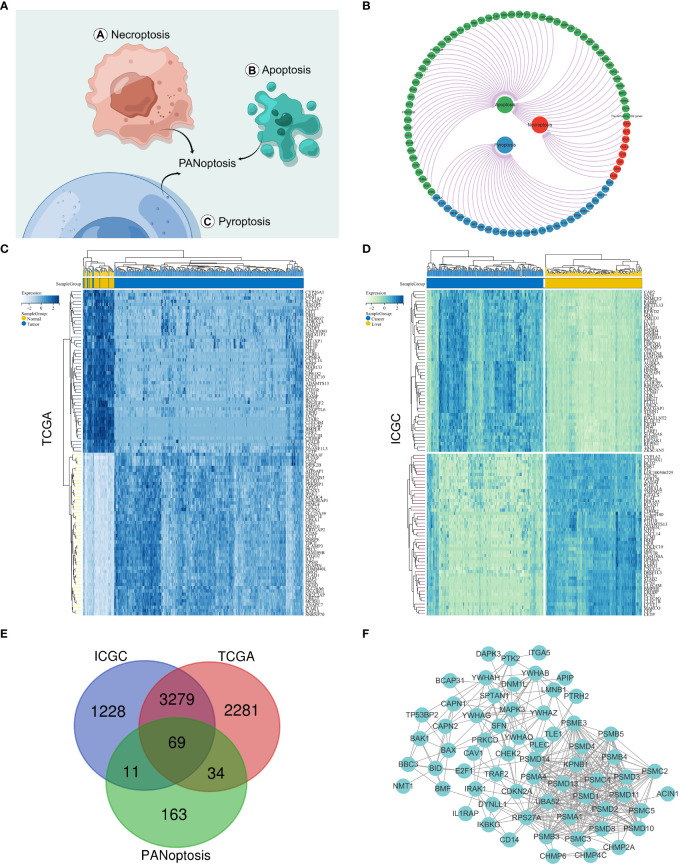
Identification of PANoptosis-associated differential genes for HCC. **(A)** Concept drawing of PANoptosis (Fig-draw website, ID: YPOYA779c7); **(B)** The PANoptosis gene list; **(C, D)** Heatmap of the top 50 up- and down-regulated DEGs between HCC and normal tissue in the TCGA and ICGC databases; **(E)** The Vene diagram is composed of the differential genes of TCGA and ICGC respectively and the PANoptosis related dataset; **(F)** Protein–protein interactions among the PANoptosis-associated differential genes for HCC (HPAN_DEGs).

The PANoptosis gene set consisted of pyroptosis (27 genes), apoptosis (259 genes), and necroptosis (15 genes) (**Figures 1A, B** and **Supplementary Figure S1A**). The differentially expressed genes for HCC comprised 5663 differentially expressed genes for hepatocellular carcinoma screened by the TCGA database and 4587 differentially expressed genes for hepatocellular carcinoma screened by the IGCG database (**Figures 1C, D** and **Supplementary Figure S1B**). We mapped the three gene sets screened for TCGA, ICGC, and PANoptosis to obtain 69 genes, defined as PANoptosis-associated differentially expressed genes for HCC (HPAN_DEGs) (**Figures 1E, F**).

**Figure 2:**
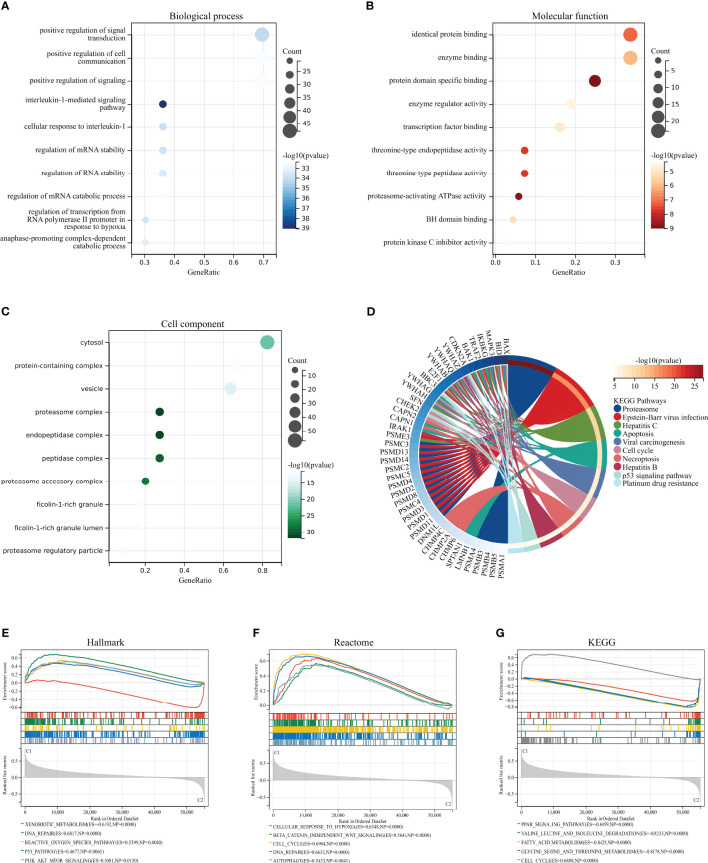
GO/KEGG/GSEA enrichment analysis of the HPAN_DEGs. **(A–C)** GO enrichment analyses based on the HPAN_DEGs; **(D)** KEGG enrichment analyses based on the HPAN_DEGs; **(E–G)** GSEA analysis based on Hallmark, Reactome and KEGG datasets respectively.

We performed GO, KEGG, and GSEA enrichment analyses to investigate the biological functions and related signaling pathways of HPAN_DEGs. Bioprocess (BP) analysis revealed that HPAN_DEGs are mainly enriched in signal transduction, cell communication, interleukin-1-mediated signaling pathway, and regulation of RNA stability (FDR<0.1, p value<0.05, **Figure 2A**). Molecular functional (MF) analysis revealed that HPAN_DEGs were mainly enriched in protein binding, enzyme binding, enzyme regulator activity, and transcription factor binding (FDR<0.1, p-value<0.05, **Figure 2B**). Cell composition (CC) analysis showed that HPAN_DEGs were mainly enriched in proteasome complex, endopeptidase complex, peptidase complex, and cytosol (FDR<0.1, p-value<0.05, **Figure 2C**). KEGG enrichment analysis suggested that HPAN_DEGs were mainly enriched in Proteasome, Apoptosis, Cell cycle, Necroptosis, p53 signaling pathway, Platinum drug resistance, and other signaling pathways (FDR<0.1, p value<0.05, **Figure 2D**).

To further clarify the biological functions undertaken by HPAN_DEGs, we conducted a GSEA analysis of HPAN_DEGs utilizing the KEGG, Hallmark, and Reactome datasets, respectively. The results showed that HPAN_DEGs were mainly enriched in the P53 signaling pathway (Hallmark and KEGG), Reactive oxygen species pathway (Hallmark), Cell cycle (Reactome and KEGG), DNA repair (Hallmark and Reactome) (NES>1, p-value <0. 05 and FDR<0.25, **Figures 2E–G**). Overall, our study identified 69 PANoptosis-associated differentially expressed genes for HCC (HPAN_DEGs) and revealed their enrichment in various biological functions and signaling pathways, such as the P53 signaling pathway, DNA repair, and cell cycle, indicating their potential involvement in tumor growth, metastasis, and drug resistance.

### HPAN_DEG expression profiling identifies three HCC subtypes with distinct prognoses

**Figure 3:**
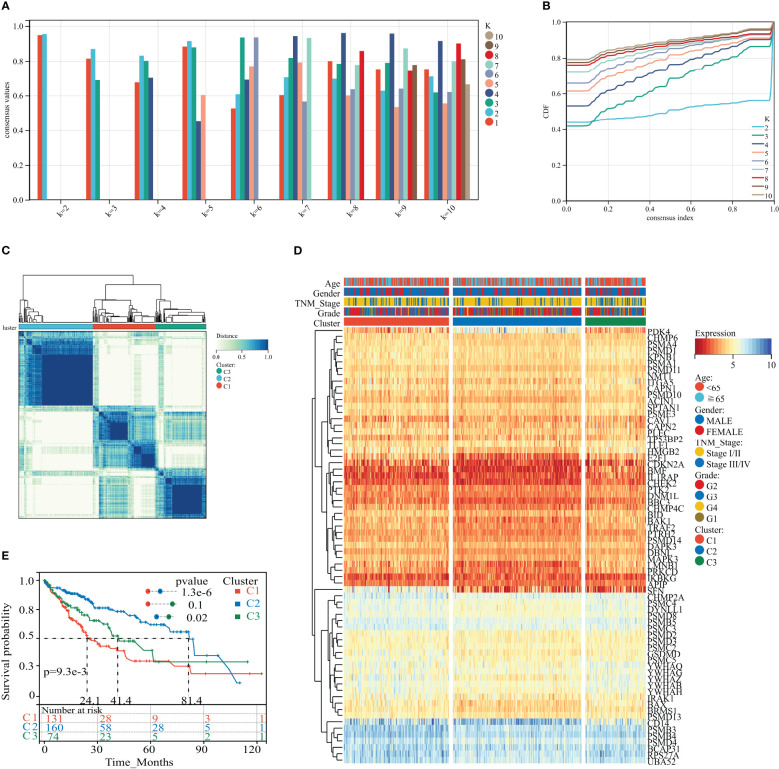
HPAN_DEG expression profiling identifies three HCC subtypes with distinct prognoses. **(A, B)** Assessment of average consistency within clusters and assessment of area under the CDF curve line when k = 2 to 10; **(C)** The training cohort was divided into three HCC subtypes by consensus clustering. **(D)** A heatmap displayed the expression of HPAN_DEGs in different HCC subtypes; **(E)** Kaplan-Meier survival analysis between three subtypes of HPAN_DEGs.

We applied consistent clustering analysis to group the HCC cohort of TCGA based on information from the expression profiles of 69 HPAN_DEGs. When the value of K was taken as 3, the average consistency within the group was higher while ensuring that the area under the CDF curve line was as large as possible (**Figures 3A, B** and **Supplementary Figure S1C**). We named cohorts Cluster_1 (n = 131), Cluster_2 (n = 160), and Cluster_3 (n = 74) (**Figure 3C**). We then compared the expression levels of PAN apoptotic genes between the three Clusters and found that the PDK4 gene was up-regulated in Cluster_2 compared to Cluster_1 and Cluster_3, while SFN expression was down-regulated in the other two groups relative to Cluster_1 (**Figure 3D** and **Supplementary Figure S1D**). Additionally, survival analysis demonstrated that these three subtypes of HPAN_DEGs exhibit distinct clinical prognostic outcomes, with Cluster_1 having the poorest overall survival rate, Cluster_2 having the best, and Cluster_3 falling in between the two (**Figure 3E**). In summary, this study applied clustering analysis to identify three distinct subtypes of HCC based on the expression profiles of 69 HPAN_DEGs, which exhibited differential expression of PAN apoptotic genes and distinct clinical prognostic outcomes.

### Distinct immunological profiles and mutational landscapes in HPAN_DEGs subgroups

**Figure 4:**
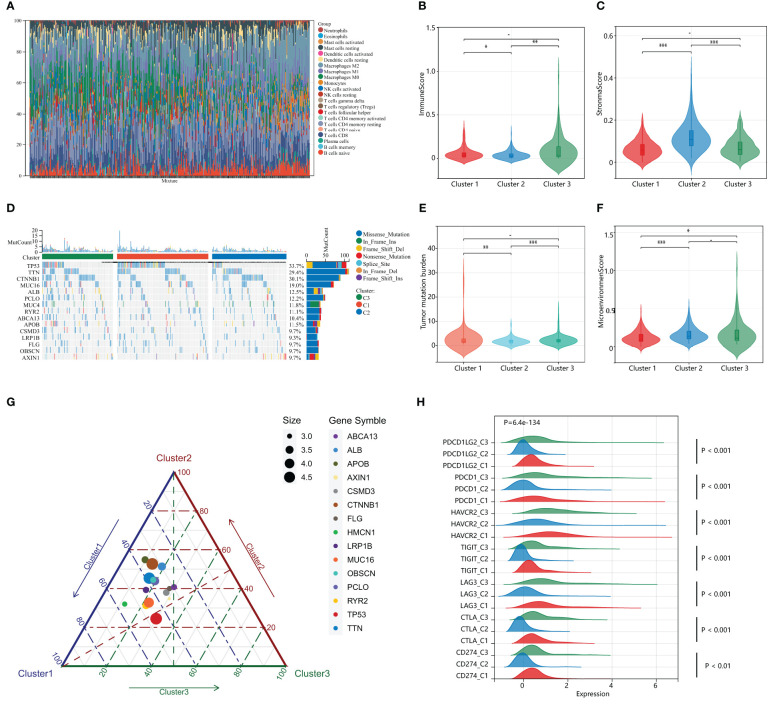
Distinct immunological profiles and mutational landscapes in HPAN_DEGs subgroups. **(A)** Waterfall diagram of the distribution of the 24 immune cells in the training set; **(B, C)** Immuno-score and Stromal-score for 3 subgroups of HPAN_DEGs. **(D)** Waterfall maps of the somatic mutations in different HPAN_DEGs subtypes; **(E)** Tumor mutation burden in different HPAN_DEGs subtypes; **(F)** Microenvironment score -score for 3 subgroups of HPAN_DEGs; **(G)** Characteristics of gene mutation in different HPAN_DEGs subtypes; **(H)** Expression of immune checkpoints in different HPAN_DEGs subtypes. *p < 0.05, **p < 0.01, ***p < 0.001.

Previous studies suggest that PANoptosis may influence tumor mutation and immune infiltration. To assess the immunological profile among subgroups of HPAN_DEGs, we performed an immunological landscape analysis of each of the three subgroups using several immunological algorithms, including CIBERSORT, ESTIMATE, and xCELL. The waterfall diagram of **Figure 4A** illustrates the distribution of the 22 immune cells in the TCGA training set. Then, we evaluated the Immune-Score, Stromal-Score, and Microenvironment-Score for HPAN_DEGs subgroups (**Figures 4B, C**). Our study indicates that Cluster_2 is significantly different from the other two groups in the term of Immune-Score and Stromal-Score (Cluster_2 vs. Cluster_1, 0.04 ± 0.05 vs. 0.06 ± 0.07, P = 0.03; Cluster_2 vs. Cluster_3, 0.04 ± 0.05 vs. 0.10 ± 0.15, P = 0.0025, Immune-Score) (Cluster_2 vs. Cluster_1, 0.12 ± 0.06 vs. 0.06 ± 0.04, P = 7.9E-19; Cluster_2 vs. Cluster_3, 0.12 ± 0.06 vs. 0.07 ± 0.05, P = 0.00000000073, Stroma-Score). In the assessment of the Microenvironment-Score, we found that Cluster_2 and Cluster_3 were not statistically different, while Cluster_1 was significantly different from the other two groups (Cluster_1 vs. Cluster_2, 0.12 ± 0.085 vs. 0.16 ± 0.09, P = 0.0000017; Cluster_1 vs. Cluster_3, 0.12 ± 0.08 vs. 0.17 ± 0.16, P = 0.03, **Figure 4F**). We also evaluated the gene expression of immune checkpoints among HPAN_DEGs subgroups, namely PD-1 (PDCD1), PD-L1 (CD274), PD-L2 (PDCD1LG2), CTLA4, LAG3, TIGIT, HAVCR2, and found that the expression of these immune checkpoints was up-regulated in Cluster_1 and Cluster_3, and downregulated in Cluster_2 (all P < 0.05, **Figure 4H**).

Subsequently, we demonstrated the somatic mutational landscape among the HPAN_DEGs subgroups. The top 15 mutated genes in the three subgroups were TP53/CTNNB1/TTN/MUC16/ALB/PCLO/MUC4/RYR2/ABCA13/APOB/CSMD3/LRP1B/FLG/OBSCN/AXIN1. The gene with the highest mutation rate was TP53, which varied among the three clusters, with Cluster_1 (44.79%) having a higher mutation rate than Cluster_2 (23.96%) and Cluster_3 (29.17%), respectively (**Figure 4D**). Tumor mutational load (TMB) is an essential indicator of the number of mutations in cancer and a novel marker for evaluating the efficacy of PD-1 antibody therapy. We compared the tumor mutational burden (TMB) of the three subgroups and found that the TMB of Cluster_2 was lower than that of Cluster_1 and Cluster_3, respectively (Cluster_2 vs. Cluster_1, 1.84 ± 1.34 vs. 2.85 ± 3.99, P = 0.0042; Cluster_2 vs. Cluster_3, 1.84 ± 1.34 vs. 2.50 ± 2.08, P = 0.00034, **Figure 4E**). We then applied a ternary diagram showing the distribution of mutant genes among different subgroups of HPAN_DEGs (**Figure 4G**). In summary, our findings demonstrated substantial discrepancies in the expression of immune checkpoints and the mutational landscape among the three clusters, which could potentially have crucial ramifications in cancer immunotherapy.

### Construction of a HPAN_DEGs-based PANoptosis risk score model for prognostic assessment in HCC

**Figure 5:**
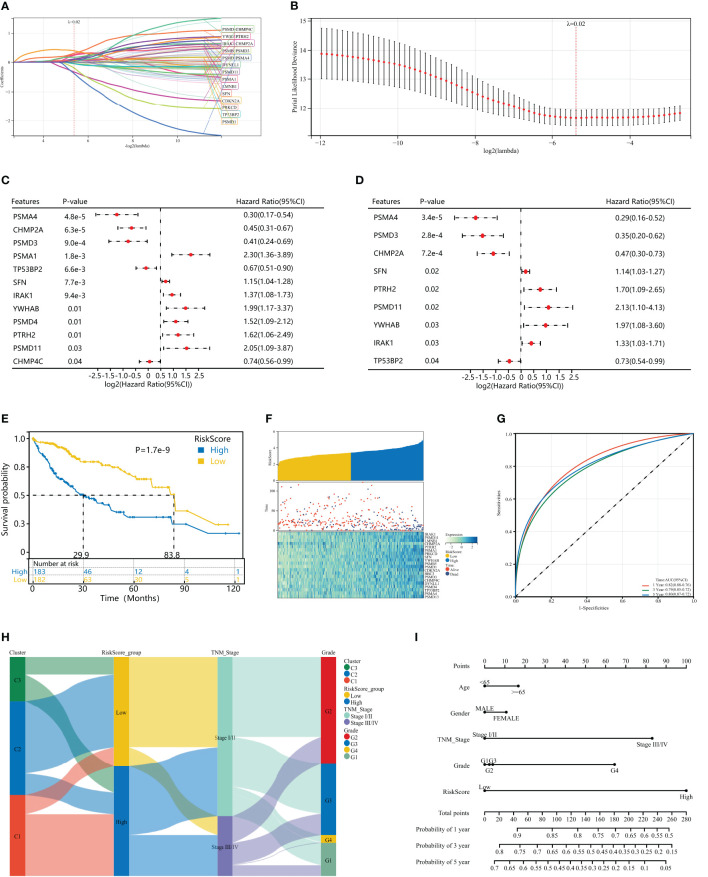
Construction of a HPAN_DEGs-based PANoptosis risk score model for prognostic assessment in HCC (HPAN-index). **(A, B)** Lasso regression analysis with 10-fold cross-validation resulted in 20 genes associated with survival (Lambda = 0.0239703477847909); **(C, D)** Univariate and multivariate Cox analyses further screened for nine PANoptosis-associated genes associated with survival; **(E)** Kaplan–Meier analyses demonstrate the prognostic significance of the HPAN-index model in the training set; **(F)** HPAN-index distribution, survival status of each patient, and heatmaps of prognostic 9-gene signature in the training set; **(G)** Receiver operator characteristic (ROC) analysis of the HPAN-index model in the training set; **(H)** Sankey diagrams shows the interrelationship between HPAN_DEGs subtypes, the risk groups of the HPAN-index and the individual clinical characteristics; **(I)** A nomogram was established to predict the prognostic of HCC patients.

To further evaluate the impact of HPAN_DEGs on survival prognosis, we used LASSO, univariate and multivariate regression to screen nine gene signatures with strong prognostic associations, and finally constructed a PANoptosis risk index for hepatocellular carcinoma (HPAN-index, **Figures 5A–D**). HPAN-index = 0.4142* IRAK1 + 0.78337*PSMD11 - 0.33085*CHMP2A + 0.66389*PTRH2 + 0.11779*SFN + 0.82753*YWHAB - 0.95811*PSMD3 - 0.25778 *TP5 3BP2 - 0.72582*PSMA4. We classified 365 patients with complete survival information in training set into high and low HPAN-index groups utilizing the HPAN-index score (high 183 vs. low 182). Kaplan-Meier analysis demonstrated a significantly better prognosis for the low-risk group (Median Survival Time, MST = 83.8 months) than the high-risk group (MST = 29.9 months) in the training set (P < 0.001, **Figure 5E**). We investigated the relationship between patient prognosis, gene expression, and HPAN-index and observed a significant decrease in survival as the HPAN-index increased. As expected, CHMP2A/PSMA4/PSMD3/TP53BP2 were protective factors, whose expression was downregulated with increasing HPAN-index, while IRAK1/PSMD11/PTRH2/SFN/YWHAB were risk factors (**Figure 5F**).

In addition, we evaluated the area under the curve (AUC) of the HPAN-index as a predictive model, and the results suggested that the HPAN-index was highly accurate in predicting survival at 1, 3, and 5 years (**Figure 5G**). We applied Sankey diagrams to visualize the relationship between the risk groups of the HPAN-index and the individual clinical characteristics, suggesting that Cluster_1 mainly converges in the high HPAN-index group. In contrast, Cluster_2 mainly converges in the low HPAN-index group (**Figure 5H**). Interestingly, Stage I/II in TNM staging mainly converged in the low HPAN-index group, while Stage III/IV mainly converged in the high HPAN-index group. We constructed a nomograph based on the Cox regression analysis results and found that the HPAN-index was an independent risk factor (**Figure 5I** and **Supplementary Figure S1E**). In conclusion, the HPAN_DEGs-based PANoptosis risk score model (HPAN-index) constructed by LASSO regression, univariate and multivariate regression analysis, can accurately predict the survival prognosis of hepatocellular carcinoma patients and could be a potential independent risk factor for clinical decision-making.

### Validation of HPAN-index as a prognostic predictor in HCC patients across multiple cohorts

**Figure 6:**
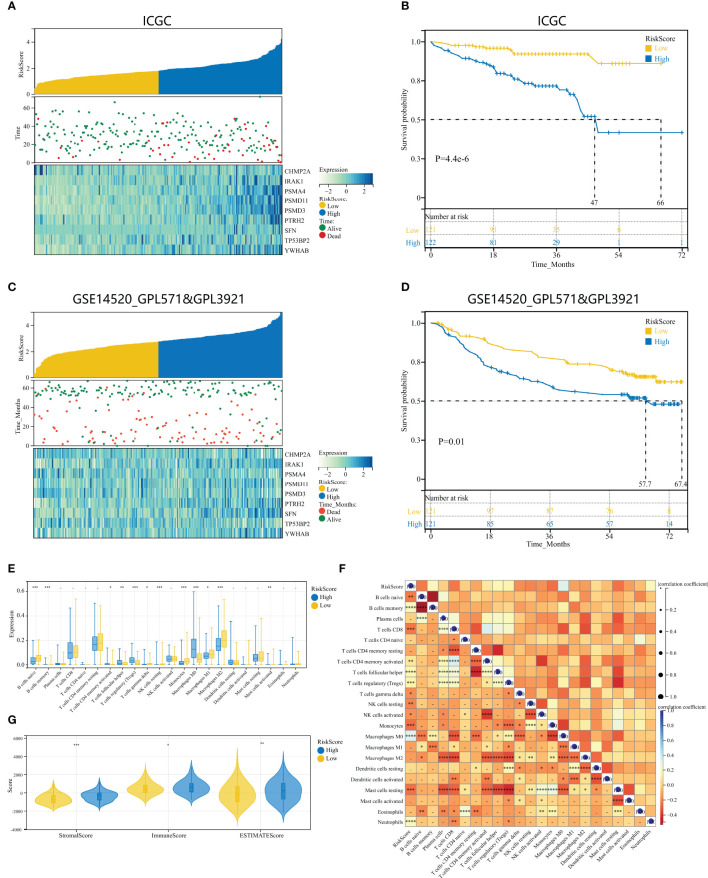
Validation of HPAN-index as a prognostic predictor in HCC patients across multiple cohorts. **(A)** HPAN-index distribution, survival status of each patient, and heatmaps of prognostic 9-gene signature in the validation set (ICGC, n = 243); **(B)** Kaplan–Meier analyses demonstrate the prognostic significance of the HPAN-index model in the validation set (ICGC, n = 243); **(C)** HPAN-index distribution, survival status of each patient, and heatmaps of prognostic 9-gene signature in the testing set (GSE14520, n = 242); **(D)** Kaplan–Meier analyses demonstrate the prognostic significance of the HPAN-index model in the validation set (GSE14520, n = 242); **(E)** Box plot visualizes significantly different immune cells between different HPAN-index groups; **(F)** Correlation of HPAN-index with immune cell infiltration evaluated using CIBERSORT in the HCC; **(G)** Immuno-score, Stromal-score, and ESTIMATE-score between different HPAN-index groups. *p < 0.05, **p < 0.01, ***p < 0.001, ****p < 0.0001.

To examine the repeatability of the model HPAN-index as a predictive model, we validated the model in the ICGC_HCC cohort (Validation set) and the GSE14520 cohort (Testing set). Applying the Kaplan-Meier analysis, we can observe a significant decrease in patient survival as the HPAN-index increases (**Figures 6A, C**). In the Validation set, the prognosis was significantly better in the low-HPAN-index group (MST = 66.7 months) than in the high-HPAN-index group (MST = 47.3 months, P < 0.001, **Figure 6B**), with similar results in the Testing set (P = 0.01, **Figure 6D**). In conclusion, the HPAN-index model demonstrated significant predictive value for patient survival in both the Validation and Testing sets, with higher HPAN-index scores indicating poorer prognosis.

### Immune cell landscape and molecular pathways associated with HPAN-index in HCC patients

**Figure 7:**
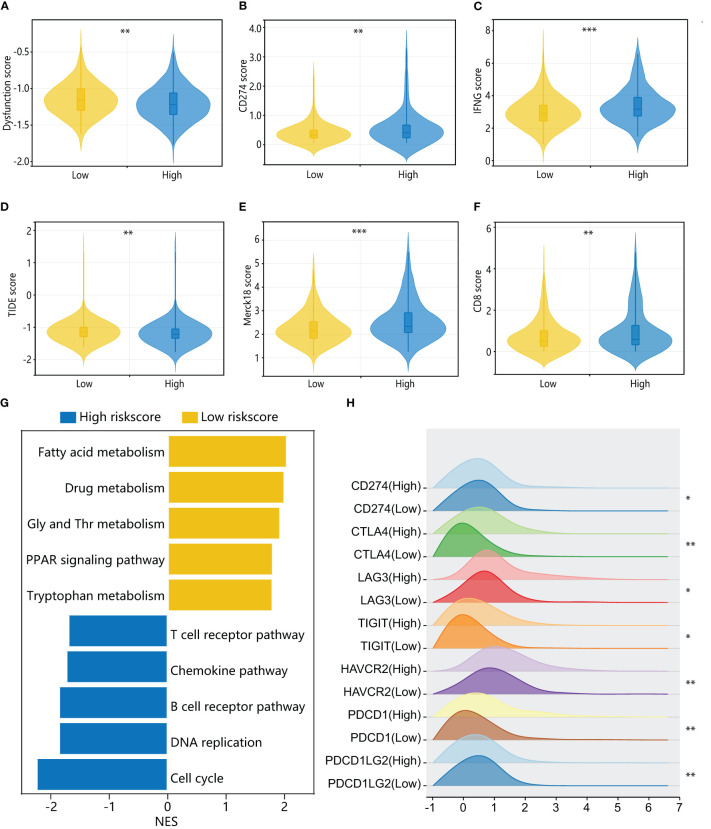
Immune cell landscape and molecular pathways associated with HPAN-index in HCC patients. **(A–F)** TIDE, immune dysfunction, immune exclusion, CD274, Merck18, CD8, and IFNγ scores in low and high HPAN-index groups; **(G)** Top enriched pathways for genes with specific expression in the high and low HPAN-index groups.; **(H)** Differential expression of immune checkpoints between the high and low HPAN-index groups. *p < 0.05, **p < 0.01, ***p < 0.001.

To further investigate the immune characteristics of different HPAN-index groups, we employed five distinct immune algorithms, including CIBERSORT, ESTIMATE, TIDE, TIMER, and xCell, to assess the relationship between HPAN-index and the immune microenvironment. The outcomes obtained from **Figures 6E, F** demonstrated a significant correlation between the HPAN-index and the expression of diverse immune cells, including B cells naive, B cells memory, T cells CD8, T cells CD4 memory activated, T cells follicular helper, T cells regulatory, T cells gamma delta, NK cells resting, NK cells activated, Monocytes, Macrophages M0, Mast cells resting, and Neutrophils, with all P values less than 0.05. Moreover, the high HPAN-index group exhibited significantly higher Stromal-Score, Immune-Score, and ESTIMATE-Score compared to the low HPAN-index group (all P<0.05, **Figure 6G**). Notably, the Microsatellite Instability (MSI)-score, an essential indicator reflecting tumor genome stability, significantly correlated with immune checkpoint efficacy (56). Based on our analysis, we observed that the high HPAN-index group has a significantly higher MSI-score compared to the low HPAN-index group, with similar results for other scores such as TIDE-score, IFNG-score, Merck18-score, Dysfunction-score, Exclusion-score, MDSC-score, and TAM M2-score (all P < 0.05, **Figures 7A–F**). Compared to the low HPAN-index group, the high HPAN-index group exhibited a significant up-regulation in the expression levels of immune checkpoints, including CD274, CTLA4, LAG3, TIGIT, HAVCR2, PDCD1, and PDCD1LG2 (all P < 0.05, **Figure 7H**). This observation suggests that the high HPAN-index group may be more likely to benefit from immunotherapy than the low HPAN-index group.

To further elucidate the molecular mechanisms underlying the different HPAN-index groups, we performed GSVA enrichment analysis. The results revealed that the high HPAN-index group was significantly enriched in signaling pathways such as the T cell receptor pathway, chemokine pathway, B cell receptor pathway, DNA replication, and cell cycle. In contrast, the low HPAN-index group showed significant enrichment in signaling pathways related to fatty acid metabolism, drug metabolism, glycine serine, threonine metabolism, PPAR signaling pathway, and tryptophan metabolism (**Figure 7G**). We further evaluated drug sensitivity in different HPAN-index groups and found that small molecule inhibitors (JAK1_8709/KRAS_G12C/Linsitinib/Nilotinib/Oxaliplatin/Niraparib/Picolinic-acid/Selumetinib/Sorafenib) showed significantly higher sensitivity in the low HPAN-index group compared to the high HPAN-index group (all P < 0.05, **Supplementary Figures S2A–I**). In summary, patients with high HPAN-index may be more responsive to immune checkpoint therapy, while those with low HPAN-index may be better suited for a targeted drug.

### Validation of HPAN-index and identification of key molecule YWHAB in sorafenib resistance

**Figure 8:**
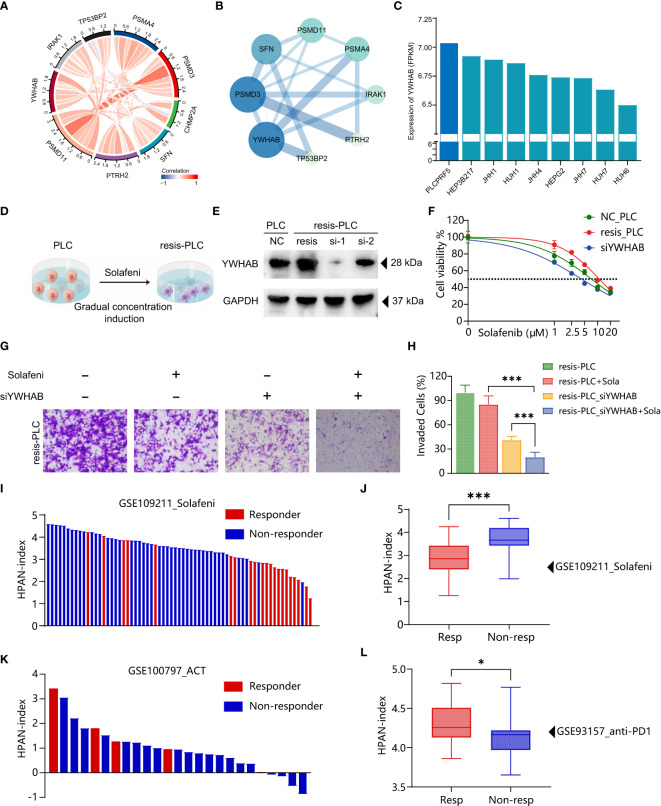
Validation of HPAN-index and identification of key molecule YWHAB in sorafenib resistance. **(A)** Chord Diagram displaying the relationship between the hub HPAN_DEGs expression in the training set; **(B)** Protein-protein interaction network diagram between the hub HPAN_DEGs; **(C)** Bar graph showing the expression of YWHAB in various hepatocellular carcinoma cell lines; **(D)** Construction of a sorafenib-resistant PLC cell line using an incremental drug concentration method (continuous induction at low and increasing concentrations); **(E)** Western blot analysis of YWHAB knockdown in sorafenib-resistant cell line (resis-PLC); **(F)** Dose-response curve of sorafenib treatment in NC_PLC, resis-PLC, and resis-PLC with YWHAB knockdown (The IC_50_ values of sorafenib for NC_PLC, resis-PLC, and siYWHAB were 7.265 μM, 11.01 μM, and 4.288 μM, respectively); **(G)** Transwell assay for evaluating the effect of YWHAB knockdown on resis-PLC cell sensitivity recovery; **(H)** Quantification of invasive cells as a percentage; **(I)** Bar graph showing the HPAN-index of different samples from GSE109211 dataset; **(J)** Box plot analysis of HPAN-index for the Sorafenib-sensitive and Sorafenib-resistant groups; **(K)** Bar graph showing the HPAN-index of different samples from GSE100797 dataset; **(L)** Box plot analysis of HPAN-index for the two groups classified as the anti-PD-1 Therapy-responsive and non-responsive groups (blue) based on the GSE93157 dataset. Data are presented as mean ± standard deviation (SD) from three independent experiments, *p < 0.05, ***p < 0.001.

To further validate the accuracy of HPAN-index in predicting drug resistance and explore the key molecules, we combined the correlation analysis results of gene expression data from TCGA and protein-protein interaction (PPI) topological network analysis results to identify YWHAB with higher weight in HPAN-index (**Figures 8A, B**). Subsequently, we utilized the DepMap database to select PLCPRF5 cells with the highest YWHAB expression levels to construct sorafenib-resistant cells (resis-PLC) (**Figures 8C, D**). We were thrilled to discover that knocking down YWHAB in resis-PLC not only restored sensitivity to sorafenib but actually resulted in even greater sensitivity than the non-resistant control (IC_50_, NC_PLC 7.265 μM, resis-PLC 11.01 μM, siYWHAB 4.288 μM, **Figures 8E, F**). As shown in **Figures 8G, H** the knockdown of YWHAB restored the ability of sorafenib to inhibit the invasion of resis-PLC. Furthermore, we further validated HPAN-index in public databases, and the results showed that the Low HPAN-index group was more sensitive to sorafenib and less responsive to immunotherapy than the High HPAN-index group (**Figures 8I–L**). In summary, the HPAN-index exhibits considerable advantages in predicting the response to immunotherapy and the sensitivity to targeted drugs, with YWHAB potentially playing a crucial role in the HPAN-index’s functionality.

## Discussion

As the clinical use of immunotherapeutic agents and molecularly targeted inhibitors continues to expand, liver cancer patients experience limited benefits compared to other malignancies such as melanoma, lung cancer, and kidney cancer (57–59). It is primarily due to the unique characteristics of the liver, which has a remarkable regenerative capacity and serves a vital role in detoxification. Furthermore, malignant tumors originating from the liver are more heterogeneous than other tumors (60). Therefore, it is of utmost importance for scholars to address the urgent question of enabling liver cancer patients to select the most appropriate clinical drugs and achieve personalized and precise treatment for liver cancer.

In recent years, scholars have proposed the concept of PANoptosis, which highlights the complex interplay between different cell death pathways in regulating tumor development (13). It is now widely acknowledged that a single death pathway does not solely govern tumor progression but involves intricate crosstalk between various pathways (14, 19). This integration of functions often has significant implications for tumor resistance and the immune microenvironment. In this study, we developed exclusive models for hepatocellular carcinoma related to PANoptosis (HPAN-index). We further validated the predictive performance of these models in terms of prognosis, minor molecule drug sensitivity, and immunotherapy in both the validation and test sets. These findings highlight the crucial role of PANoptosis in the context of hepatocellular carcinoma and offer valuable insights for developing personalized and precise therapeutic strategies.

The team of Prof. Gao Q. identified three distinct subtypes of hepatocellular carcinoma (HCC): the Metabolic subtype, the Proliferative subtype, and the Tumor Microenvironment Dysregulation subtype (60). Our study conducted a consistency clustering analysis of 69 HPAN_DEGs in the training set and identified three subgroups that exhibit distinct characteristics regarding overall survival prognosis, mutational landscape, and immune infiltration. Cluster_1, which demonstrated the highest tumor mutational load, had the worst overall survival rate. However, Cluster_1 showed an advantage in immunotherapy with higher expression of its major immune checkpoints CD274/CTLA4/LAG3/TIGHT/HAVCR2/PDCD1/PDCD1LG2 compared to the other groups. On the other hand, Cluster_2 presented an opposite phenotype in terms of overall survival prognosis, mutational landscape, and immunomolecular profile compared to Cluster_1. We observed that Cluster_1 presented SFN+PDK4-, whereas Cluster_2 presented SFN-PDK4+. Our molecular characterization of these three subgroups revealed essential insights into the complex interplay between tumor mutational load, immune checkpoint expression, and prognosis, providing valuable information for developing personalized therapeutic strategies for hepatocellular carcinoma.

Previous studies have demonstrated that SFN is an oncogene, accelerating tumorigenesis and progression across various cancer types (61, 62). Prof. Masayuki Noguchi’s team identified that SFN specifically binds to ubiquitinated protease 8 (USP8) in lung adenocarcinoma cells, enhancing the stabilization of receptor tyrosine kinases (RTKs), including EGFR and MET, through abnormal regulation of USP8. These findings suggest that SFN may be a promising therapeutic target for lung adenocarcinoma. In line with this, our study also found that positive expression of SFN in Cluster_1 could indicate the potential for tumor proliferation in these patients. Pyruvate dehydrogenase kinase 4 (PDK4) encodes an enzyme that regulates cellular metabolism by inhibiting the phosphorylation of a key regulatory enzyme of glucose oxidation, pyruvate dehydrogenase complex (PDC) (63). High expression of PDK4 has been associated with altered metabolic pathways in tumor cells, including lactic acidification and malignant transformation (64–66). In our study, the positive expression of PDK4 in Cluster_2 may suggest that the tumor type in these patients is associated with aberrant tumor cell metabolism. Overall, our study identified three subgroups based on HPAN_DEGs with distinct characteristics in terms of prognosis, mutational landscape, and immune infiltration. Furthermore, these subgroups exhibited differential expression of SFN and PDK4 genes. Our findings contribute to a better understanding of the biology of these tumor types and may provide a new basis for subgroup screening in hepatocellular carcinoma.

Using LASSO regression and univariate and multifactorial analyses, we screened nine genes strongly associated with prognosis and constructed the HPAN-index. With this model, we divide the cohort into High-index and Low-index groups, where the High-index group is mainly from Cluster_1, and the Low-index group is mainly from Cluster_2. We note that the High-index group showed a significant immune activation status. In contrast, the Low-index group showed a significant advantage in sensitivity to small molecule targeted drugs, which is consistent with Yutian Zou et al. (67). We found that the High-index group is mainly enriched in T cell receptor pathways, B cell receptor pathways, Chemokine pathways, DNA replication, and Cell cycle pathways. Professor Peter P. Lee’s research has shown that T/B receptor pathways are closely related to T cell activation and affect PD-1 expression on T cells (68). Combined with the higher expression of PANoptosis-related genes in the High-index group, we suggest that this immune activation state in the High-index group may be related to PANoptosis. Also of note, the Low-index group demonstrated significant sensitivity to some small molecule-targeted drugs, such as sorafenib, a first-line agent for the treatment of advanced primary liver cancer (69). It may be because the Low-index group is mainly enriched in Fatty acid metabolism, Drug metabolism, PPAR signaling pathway, Tryptophan metabolism, and other pathways closely related to tumor growth, metabolism, and drug resistance (70, 71). Overall, the HPAN-index may serve as an independent risk factor for predicting the prognosis of patients with hepatocellular carcinoma and as a strategy for selecting patients for immunotherapy and targeted therapeutic agents.

Apoptosis is one of the crucial mechanisms underlying tumor cell drug resistance (72). YWHAB is a gene in the human genome that encodes the 14-3-3 protein beta/alpha. The 14-3-3 protein family is a highly conserved group of molecular chaperones that participate in various cellular signaling and regulatory processes, such as metabolism, protein transport, signal transduction, apoptosis, and cell cycle (73). Silencing of YWHAB can increase the translocation of B-cell lymphoma 2 (BCL-2)-associated death promoter (BAD) from the cytoplasm to the mitochondria, thereby inducing cell apoptosis (74). The BCL-2 family is a critical group of molecules in the field of tumor drug resistance, and its mechanism of inducing drug resistance is mainly achieved by inhibiting the apoptotic pathway of tumor cells. Studies have shown that the ratio of BCL-2/Bax is higher in drug-resistant cells than in sensitive cells (75). YWHAB, as an anti-apoptotic protein, can also cause insulin resistance in cells by affecting mitochondrial polarization (76). Our research suggests that YWHAB may play an essential role in affecting cell drug sensitivity, providing insights to further study the mechanisms of drug resistance and develop new therapeutic strategies.

There are limitations to our study that should be acknowledged. We lack the necessary single-cell level sequencing data and spatial transcriptome data to comprehensively support our analysis of the immune landscape of hepatocellular carcinoma. As the immune microenvironment is a complex microscopic system, the information on the differences and interactions between cells is inevitably lost through macroscopic bulk-RNA-seq data analysis in isolation. Moreover, a large sample size of immunotherapy-related data for hepatocellular carcinoma is needed to validate the model. Therefore, we will seek to obtain such data to validate the model further. We take this opportunity to call on the scientific community to share data related to immunotherapy for liver cancer, thereby advancing the scientific understanding of this complex disease.

## Conclusions

In conclusion, we screened for PANoptosis-associated differentially expressed genes (HPAN_DEGs) in hepatocellular carcinoma, which allowed us to identify three subgroups that exhibit distinct characteristics in terms of prognosis, mutational landscape, and immune infiltration. These subgroups also exhibited differential expression of SFN and PDK4, which may contribute to a better understanding of the biology underlying hepatocellular carcinoma. Additionally, we developed the HPAN-index, which is highly correlated with survival prognosis, sensitivity to small molecule-targeted drugs, and response to immunotherapy. We hope that applying this model will enable the identification of individuals more suitable for either immunotherapy or targeted therapy. This study provides a new strategy for the personalized and precise treatment of HCC and may shed light on future investigations into the mechanisms of PANoptosis in this disease.

## Data availability statement

The datasets presented in this study can be found in online repositories. The names of the repository/repositories and accession number(s) can be found within the article/**Supplementary Material**.

## Author contributions

FS performed the research and wrote the paper. FS, C-GW, and X-LL performed the data collection and normalization. C-GW and J-ZM carried out the *in vitro* validation. C-WH and T-LW participated in the coordination of the research. FS, YZ, and LH performed the statistical analysis. FS and ZC participated in the study design. ZC edited the manuscript. All authors read and approved the final manuscript.

## Supplementary material

Supplementary Figure 1
**(A)**. Venn diagram illustrating the overlap between gene sets related to pyroptosis, apoptosis, and necroptosis; **(B)**. Volcano plot displaying differentially expressed genes in hepatocellular carcinoma (HCC) derived from TCGA and ICGC databases; **(C)**. Heatmap demonstrating sample clustering consistency; **(D)**. Graphical representation of the proportion of SFN+ PDK4- and SFN-PDK4+ in three HPAN_DEGs subgroups; **(E)**. A prognostic nomogram based on HPAN-index gene signatures was developed to predict the outcomes of HCC patients.

Supplementary Figure 2 Efficacy of HPAN-index in predicting drug sensitivity **(A-I)** Box plots illustrating the comparison of IC_50_ values for various drugs between the high-HPAN-index group (depicted in red) and the low-HPAN-index group (depicted in green). P-values are presented in scientific notation for each comparison.
