# Supplementary figures and images for: PANoptosis-based molecular subtyping and HPAN-index predicts therapeutic response and survival in hepatocellular carcinoma
Source: Front Immunol. 2023 Jun 15;14:1197152. doi: 10.3389/fimmu.2023.1197152 (PMC10311484; doi:10.3389/fimmu.2023.1197152)

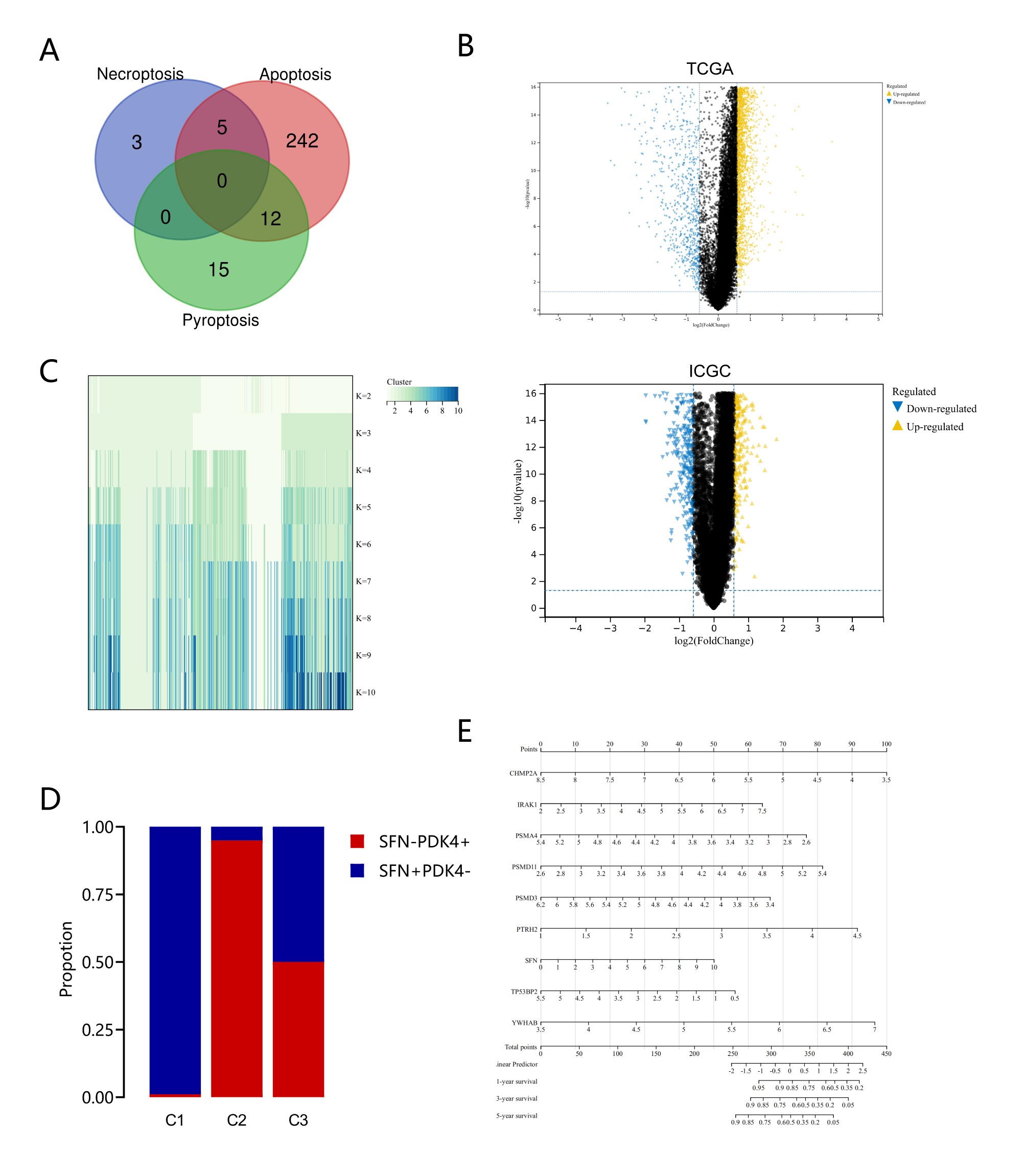

Supplement: Supplementary Figure 1 — (A). Venn diagram illustrating the overlap between gene sets related to pyroptosis, apoptosis, and necroptosis; (B). Volcano plot displaying differentially expressed genes in hepatocellular carcinoma (HCC) derived from TCGA and ICGC databases; (C). Heatmap demonstrating sample clustering consistency; (D). Graphical representation of the proportion of SFN+ PDK4- and SFN-PDK4+ in three HPAN_DEGs subgroups; (E). A prognostic nomogram based on HPAN-index gene signatures was developed to predict the outcomes of HCC patients. [file Image_1.tif]

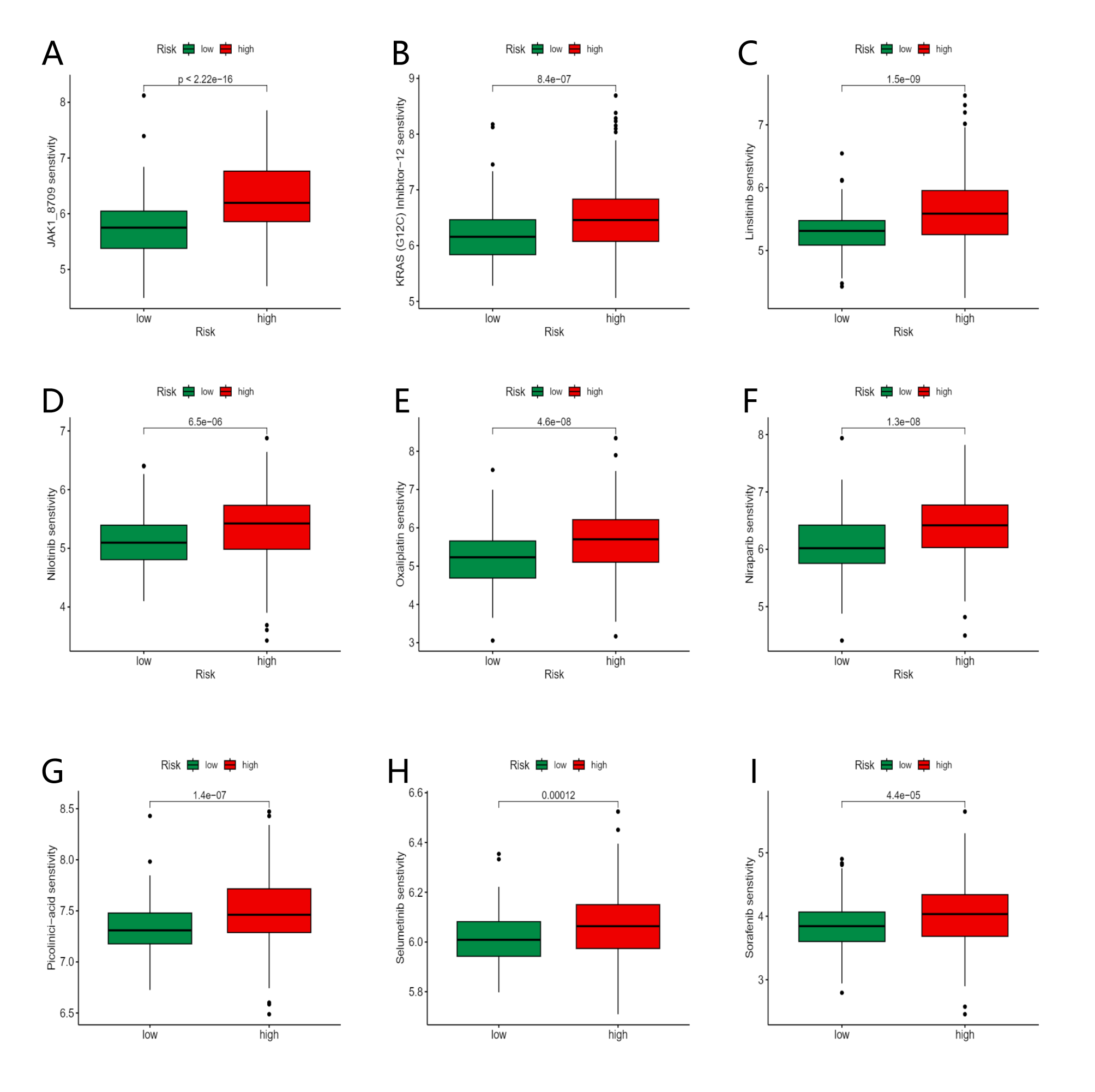

Supplement: Supplementary Figure 2 — Efficacy of HPAN-index in predicting drug sensitivity (A-I) Box plots illustrating the comparison of IC50 values for various drugs between the high-HPAN-index group (depicted in red) and the low-HPAN-index group (depicted in green). P-values are presented in scientific notation for each comparison. [file Image_2.tif]
